# Medical Items

**Published:** 1893-03

**Authors:** 


### *MEDICAL ITEMS.*

---

Dr. M. B. Hutchins will hereafter include Diseases of the Genito-Urinary Organs in his practice, in addition to Diseases of the Skin.

---

Our friend Dr. R. E. L. Barnum, formerly of Richland, Ga., has removed to Savannah, Ga., number 212 Hull St., where he will hereafter practice his profession.

---

On Wednesday, February 22d, Dr. Max Jackson was married to Miss Bertha Nussbaum, both of Macon, Ga. Dr. and Mrs. Jackson will accept The Journal's hearty good wishes.

---

The friends of Dr. Roberts Bartholow, and the medical profession generally, will be pleased to know that his health is now entirely restored, and that he has resumed his practice in Philadelphia.

---

The prize of one hundred dollars, offered by Dr. Geo. M. Gould for the best essay on Homeopathy, has been awarded to Dr. William W. Browning, of Brooklyn. There were thirteen competitors.

---

It is reported that a fad of Boston society is to intoxicate themselves with compound oxygen. It is stated that the intoxication produced by the oxygen is far less deleterious than that caused by Browning.—*Ex.*

THERE will be no patent nostrums at the World's Fair. A pamphlet issued by the Columbian Exposition says:

"Articles that are in any way dangerous or offensive, also patent medicines, nostrums and empirical preparations whose ingredients are concealed, will not be admitted to the Exposition."

---

The doctor who ignores the medical press is a drone in the medical hive. He is not a safe practitioner, nor a fit associate for progressive men. The doctor who never reads a medical journal never drives a fat horse, never wears good clothes, nor makes a mark in the world.—*Cal. Med. Jour.*

---

THE third Congress of American Physicians and Surgeons will be held in Washington May, 1894. Officers: Dr. A. L. Loomis, of New York, President; Dr. W. H. Carmalt, New Haven, Secretary; Dr. John S. Billings, Treasurer; Dr. S. C. Busey, Washington, Chairman Committee of Arrangements.

---

THE North Carolina *Medical Journal* came out in January in a new dress and under a new management. It is now a 48-page, double column journal, and its appearance is much improved. Drs. R. S. Jewett and J. A. Hodges are the new editors and proprietors. The new management makes a very good beginning, and we wish it continuous and increasing success.

---

The Rush Medical College and the College of Physicians and Surgeons, Chicago, have offered their entire properties to the Chicago University, and the faculties of the colleges have offered to resign unconditionally, in order to establish a medical department of equal grade with the other departments of this well organized university. A million dollars are said to be in sight to endow the new arrangement. This example might well be followed outside of Chicago.

---

THE International Congress of Charities, Correction and Philanthrophy will meet in the city of Chicago on Monday morning, June 12, 1893, at 10 o'clock, in a hall to be hereafter announced. The object of the International Congress of Charities, Correction and Philanthrophy is to bring together in the city of Chicago during the time of the World's Columbian Exposition interested persons of all countries to discuss matters charitable, correctional and philanthropic.

---

A NEW medical newspaper is being issued from Chicago, called *The Medical Bulletin.* It is to be published every Saturday, and will contain only medical news, with perhaps a few abstracts from other journals. It will devote special attention to medical matters connected with the World's Fair. A list of all the physicians arriving in the city will be given. Physicians will be helped in finding lodging places, and rooms will be set aside which all physicians are invited to use, where information can be obtained and mail received.—*Ex.*

---

RECENTLY when the Hobart estate was being settled in court, Dr. C. N. Ellinwood, of San Francisco, presented a claim for $30,000 for professional services rendered the deceased millionaire and his wife during their lifetime. The bill was in proper form and was approved by all the parties concerned. Judge Levy, who says he has had no personal experience with the charges of physicians for professional services, however, thought the amount was unreasonable and excessive, and without further ceremony cut it down to $10,000. This unwarrantable proceeding has naturally aroused the attention of the profession, and its termination will be awaited with much interest.—*Occidental Med. Times.*

